# Transcriptome profiling of developing testes and spermatogenesis in the Mongolian horse

**DOI:** 10.1186/s12863-020-00843-5

**Published:** 2020-04-28

**Authors:** Bei Li, Xiaolong He, Yiping Zhao, Dongyi Bai, Ming Du, Lianjie Song, Zhuang Liu, Zhenchen Yin, Dugarjaviin Manglai

**Affiliations:** 1 College of Animal Science, Equine Research Center, Inner Mongolia Agricultural University, Hohhot, 010018 China; 2 lnner Mongolia Key Laboratory of Equine Genetics, Breeding and Reproduction, Equine Research Center, Inner Mongolia Agricultural University, Hohhot, 010018 China; 3 Scientific Observing and Experimental Station of Equine Genetics, Breeding and Reproduction, Ministry of Agriculture and Rural Affairs, Equine Research Center, Inner Mongolia Agricultural University, Hohhot, 010018 China; 4 Inner Mongolia Academy of Agricultural and Animal Husbandry Sciences, Hohhot, 010031 China

**Keywords:** Mongolian horse, Developing testis, Spermatogenesis, RNA-Seq

## Abstract

**Background:**

Horse testis development and spermatogenesis are complex physiological processes.

**Methods:**

To study these processes, three immature and three mature testes were collected from the Mongolian horse, and six libraries were established using high-throughput RNA sequencing technology (RNA-Seq) to screen for genes related to testis development and spermatogenesis.

**Results:**

A total of 16,237 upregulated genes and 8,641 downregulated genes were detected in the testis of the Mongolian horse. These genes play important roles in different developmental stages of spermatogenesis and testicular development. Five genes with alternative splicing events that may influence spermatogenesis and development of the testis were detected. GO (Gene ontology) and KEGG (Kyoto Encyclopedia of Genes and Genomes) pathway analyses were performed for functional annotation of the differentially expressed genes. Pathways related to “spermatogenesis,” male gamete generation,” “spermatid development” and “oocyte meiosis” were significantly involved in different stages of testis development and spermatogenesis.

**Conclusion:**

Genes, pathways and alternative splicing events were identified with inferred functions in the process of spermatogenesis in the Mongolian horse. The identification of these differentially expressed genetic signatures improves our understanding of horse testis development and spermatogenesis.

## Background

The mammalian testis is an important male reproductive organ that produces sperm and androgens. Spermatogenesis is a complex developmental process with a unique mechanism of cell division—meiosis—as a major defining event. Sperm development consists of three stages: (1) proliferation and differentiation of spermatogonia, (2) meiosis of spermatocytes, and (3) sperm maturation. Spermatogonial stem cells differentiate into spermatogonia and, with replication of their DNA content, give rise to primary spermatocytes. Secondary spermatocytes and spermatids are derived from the primary spermatocytes by DNA replication and tetraploid meiosis. Haploid sperm cells undergo at least four distinct morphological changes into sperm cells, namely chromatin condensation, acrosome formation, flagellar formation, and cytoplasmic reduction. In addition to spermatogenic cells, spermatogenesis also involves multiple somatic cells in testicular tissues, such as Sertoli and Leydig cells. In mammalian testes, spermatogenesis occurs within seminiferous tubules, where germ cells associate with Sertoli cells, which provide the appropriate microenvironment for spermatogenesis. Related genes in these cells play important roles in specific stages of germ cell development, and the transcription and translation of these genes vary in different stages of spermatogenesis [1, 2].

RNA sequencing technology (RNA-Seq) technology, which provides a global view of gene expression profiles, has been developed for mapping and quantifying transcriptomes [3, 4]. This methodology has several advantages over other transcriptome technologies, such as higher resolution and sensitivity, a large dynamic range of gene expression levels, and the ability to identify novel transcribed regions and splicing isoforms of known genes [5]. Ramsköld [6] assessed different mammalian tissues by RNA-Seq and found that the vast majority of genes are specifically expressed in the testis. Furthermore, Djureinovic [7] categorized 20,050 putative human genes based on RNA-Seq expression patterns. These genes were specifically expressed in normal human testicular tissues compared to 26 other normal human tissue types in seven individuals. The analysis showed that the testis has the highest number of tissue-specific genes by far. Anand [8] identified differentially expressed genes (DEGs) in the human testis relative to other tissues by microarray analysis, and a total of 2,868 upregulated and 2,011 downregulated transcripts were identified. The testis thus seems to be highly metabolically active relative to other normal tissues as indicated by functional annotation. Currently, most of the studies on the relationship between the testis development and spermatogenesis have focused on humans or mice. However, little is known regarding the spermatogenesis process in the horse.

The Mongolian horse is one of the most famous native breeds in China, and in the world. It is a dual-purpose breed that can adapt to harsh climate and extensive breeding conditions. Normal development of the testis and spermatogenesis is extremely important to ensure high level semen production and protect population continuity, and genes that are important for different stages of testis development and spermatogenesis are likely to have roles in maintaining fertility. Unfortunately, the transcriptional expression of genes during testicular development and spermatogenesis in the horse is not well understood, thereby limiting the understanding of the mechanisms of deformation and maturation in spermatogenesis. This study aimed to study the transcriptome profiles of immature and mature male Mongolian horse testes utilizing high-throughput RNA-Seq technology and bioinformatics analysis. For this purpose, a testis transcript database was obtained, and DEGs and regulatory pathways were analyzed. This study provides new insights into gene expression and the regulatory mechanisms of horse testis development and spermatogenesis.

## Results

### mRNA expression profiles in Mongolian horse testis development

To screen for genes related to testis development and spermiogenesis, a total of six libraries—three from immature colts (BSM1, BSM2, and BSM3) and three from mature stallions (ASM1, ASM2, and ASM3)—were constructed and sequenced on the Illumina Hiseq platform. An overview of the information for these libraries is listed in Table S1. The libraries were found to contain 58,277,598 (BSM1), 59,921,568 (BSM2), 53,371,478 (BSM3), 59,523,578 (ASM1), 61,727,216 (ASM2), and 53,956,064 (ASM3) raw reads. To ensure the quality of subsequent analysis, the adapter reads, the N base > 10% and low-quality reads were removed; the results were 57,482,858 (BSM1), 58,972,988 (BSM2), 52,475,320 (BSM3), 58,411,056 (ASM1), 60,775,868 (ASM2), and 53,135,736 (ASM3) clean reads. The error rate and GC content of each library were calculated to control for the quality of libraries (Table S1). These results verify that the six libraries were of high quality.

The clean reads were aligned to the reference genome of *E. caballus*, which is the most accurate and commonly used reference genome for horses [9, 10], using TopHat v2.0.12. In total, 84.85% (BSM1), 85.05% (BSM2), 84.25% (BSM3), 84.38% (ASM1), 84.14% (ASM2), and 84.23% (ASM3) clean reads were uniquely mapped to the reference genome. In addition, more than 40% of clean reads were non-splice reads in each sample. Additionally, 27.98% (BSM1), 27.35% (BSM2), 26.92% (BSM3), 36.17% (ASM1), 34.89% (ASM2), and 34.74% (ASM3) of the clean reads were mapped to the borders of exons (also called “junction reads”) (Table S2). All RNA-Seq data have been submitted to The Gene Expression Omnibus, accession number GEO: GSE101697.

### Analysis of alternative splicing

Alternative splicing (AS) is a common occurrence in most eukaryotic cells and can result in the translation of different forms of proteins at various time points and environments, which increases the adaptability and physical fitness of the species. In our study, the AS events were classified into five kinds by rMATS (http://rnaseq-mats.sourceforge.net/index.html): (1) SE: Skipped exon, (2) MXE: Mutually exclusive exon, (3) A5SS: Alternative 5′ splice site, (4) A3SS: Alternative 3′ splice site, and (5) RI: Retained intron. The kinds and quantities of AS events were counted, and the expression of each type of AS event was analyzed. The results indicate that numerous and varied AS events exist in the spermatogenesis process (Table S3).

### Analysis of DEGs

**Fig. 1:**
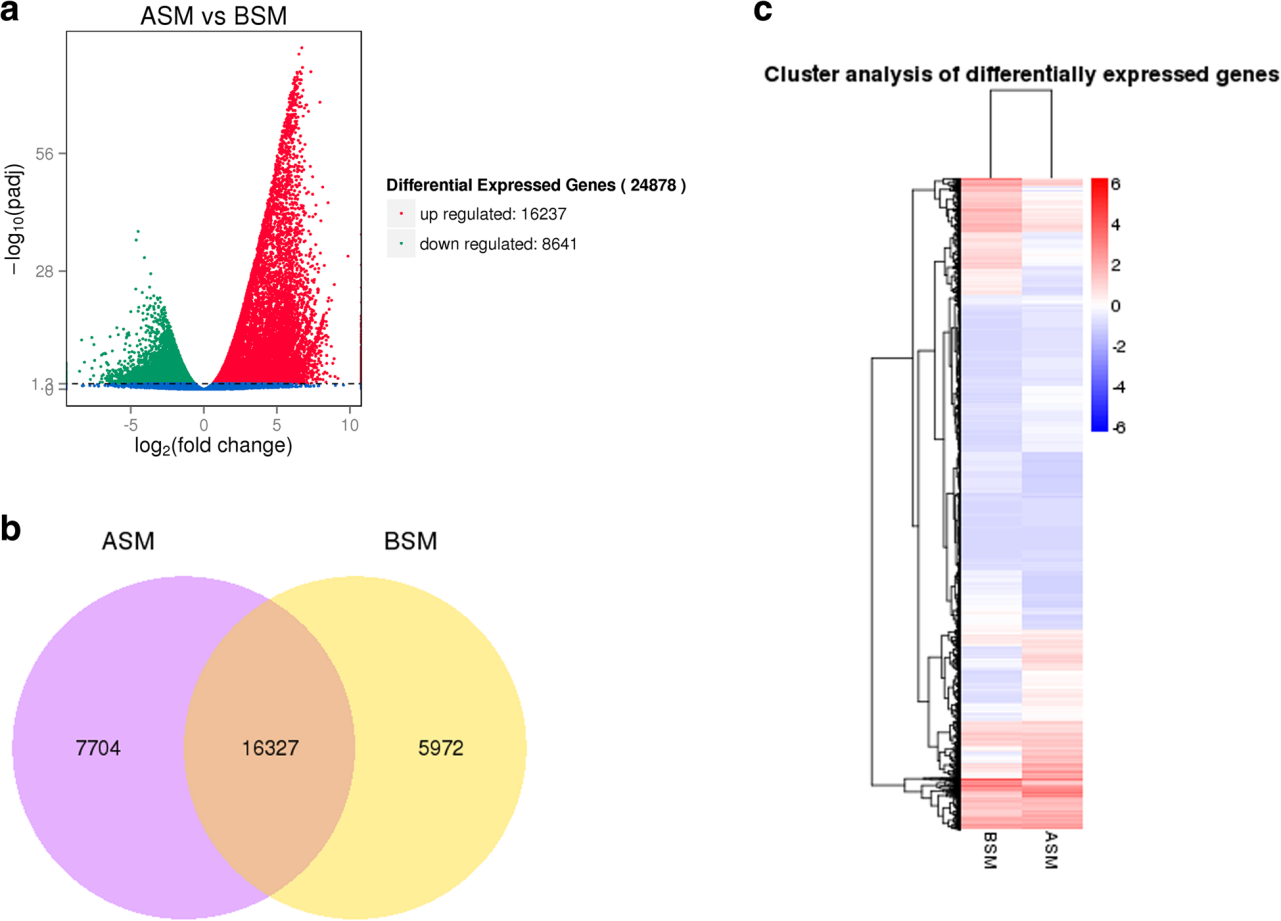
**a** Volcanic map of differentially expressed genes. There were significant differences in the expression of genes indicated by red (up) and green (down) regulation in before sexual maturation (BSM) and after sexual maturation (ASM) samples. No significant differences were observed in the expression of genes indicated by blue. The abscissa represents the fold change of genes in different samples; the ordinate represents the significant statistical difference of gene expression changes. **b** Venn diagram of gene expression. The sum of the numbers in each large circle represents the total number of genes expressed by each group, and the overlapping part of the circle represents the commonly expressed genes between groups with FPKM > 1. **c** Differential gene cluster. Overall FPKM hierarchical clustering diagram is based on the log_10_ (FPKM + 1) value for the normalization of the conversion (scale number) and clustering. Red represents high expression genes, and blue represents low expression genes

The DEGs between libraries were screened based on DESeq analysis, taking padj < 0.05 as the cut-off. By considering libraries, the 16,237 up- and 8,641 down-regulated genes were identified in total that were up-regulated in ASM versus BSM (Fig. 1a). Venn diagrams were constructed to summarize the data, which show that the ASM and BSM libraries shared 16,327 genes that were expressed in both BSM and ASM (Fig. 1b). Figure 1c shows the results of hierarchical clustering, with the DEGs of the six libraries divided into two clusters. These results show that the ASM and BSM libraries had overall distinct patterns of differential expression but that the replicates within each developmental stage were similar.

For further assessment of DEGs that are transcription factors. A total 922 transcription factors belong to 60 transcription factors families.

### GO enrichment analysis of DEGs

**Fig. 2:**
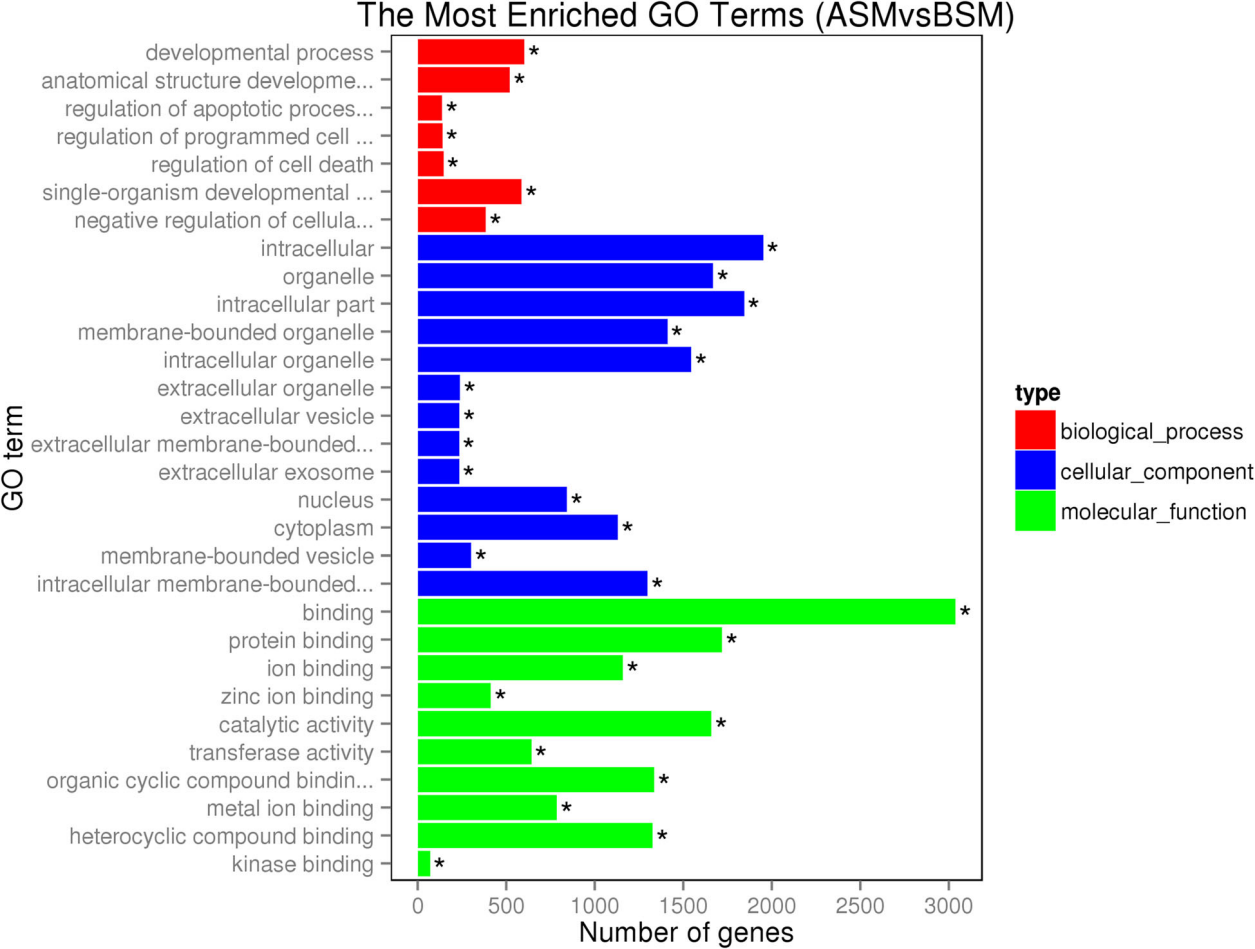
The most highly enriched GO terms. The ordinate represents the enriched GO terms, and the abscissa represents the number of differential genes. Different colors were used to distinguish “biological processes,” “cellular components” and “molecular functions.” “*” indicates significant enrichment of GO terms

Gene ontology (GO) enrichment analysis was used to explore the functions of the DEGs in testis development. A total of 70 GO terms related to “biological processes,” “molecular functions,” and “cellular components” were significantly enriched across the ASM vs BSM. DEG groups (Fig. 2). Approximately 32 GO terms belong to “biological processes,” and these were mainly focused on “metabolic process” (2,454 genes), “organic substance metabolic process” (2,044 genes), and “cellular metabolic process” (1,948 genes). Approximately 20 GO terms belong to the functional category of “cellular component,” and these were mainly concentrated in “intracellular” (1,951 genes), “intracellular part” (1,844 genes), and “organelle” (1,668 genes) classification groups. Approximately 18 GO terms belong to “molecular function,” which centered on “binding” (3,037 genes), “protein binding” (1,719 genes), and “catalytic activity” (1,659 genes).

### KEGG pathway enrichment analysis of DEGs

**Fig. 3:**
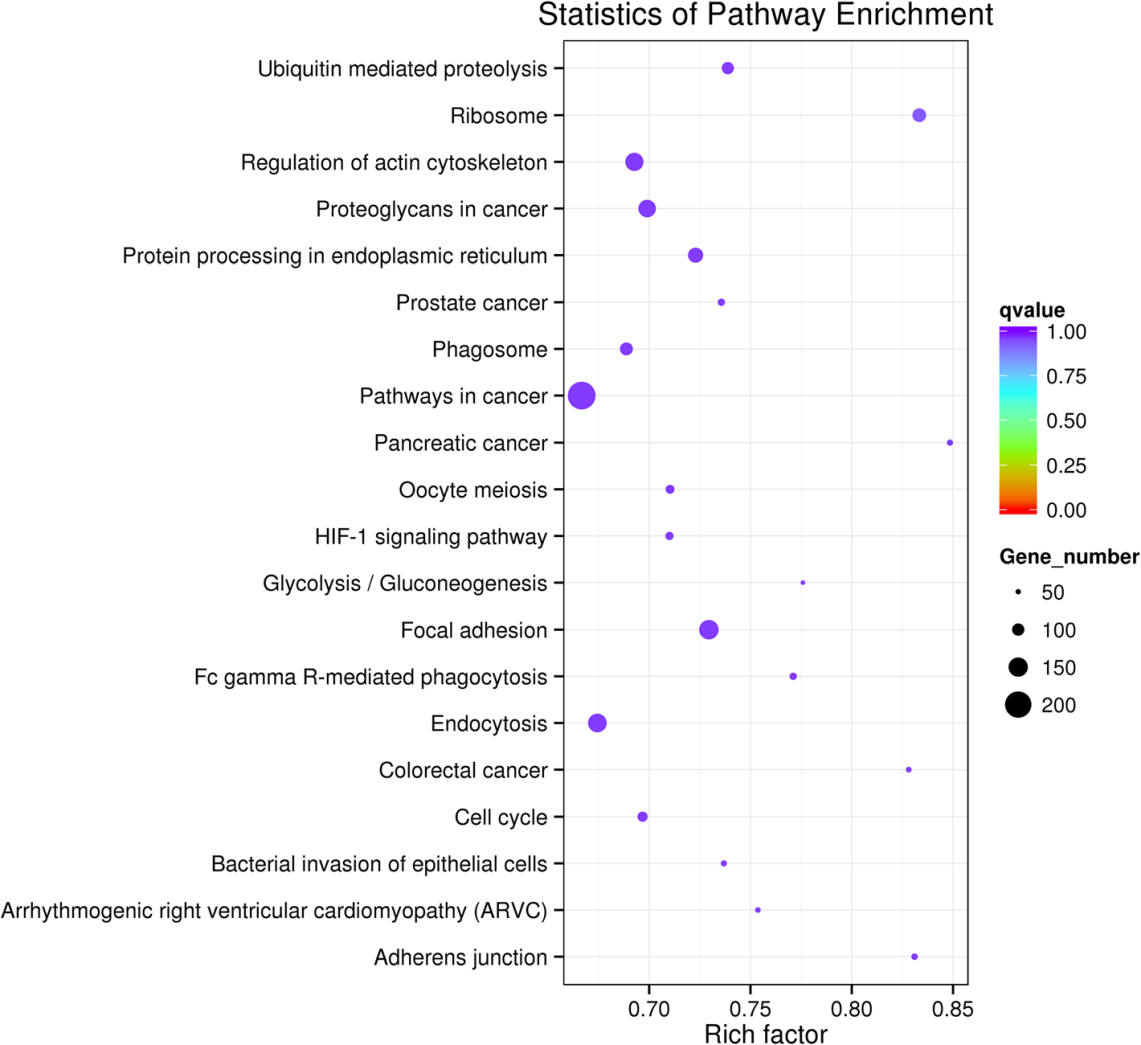
Scatter plot of differentially expressed KEGG genes. Note: The vertical axis represents the name of the pathway; the horizontal axis represents the Rich factor. The differential size of the points represents the number of differential expression genes, and the color of points signifies different ranges of Q values

Kyoto Encyclopedia of Genes and Genomes (KEGG) pathway enrichment analysis was performed on these differently expressed genes with a cut-off criterion of corrected *P*-values < 0.05. Six KEGG pathways were upregulated, including “ubiquitin-mediated proteolysis,” “protein processing in endoplasmic reticulum,” “RNA transport,” “metabolic pathways,” “oocyte meiosis,” and “cell cycle.” Four pathways were downregulated, including “ribosome,” “focal adhesion,” ‘proteoglycans in cancer,” and “ECM-receptor interaction” (Fig. 3).

### Quantitative real time PCR (qRT-PCR) validation of DEGs

To verify the differential expression of genes in ASM vs. BSM testis, we selected eight DEGs, including *MEI1* (two different sites), *EHMT2*, *BTRC*, *CLOCK*, *IQCG*, *ANAPC5* and *SKP1,* to validate the expression patterns by qRT-PCR. These genes belong to “oocyte meiosis” KEGG pathways and GO terms “spermatogenesis,” “male gamete generation,” and “spermatid development” (Table S4). There was a relatively high correlation between the mRNA expression levels of the eight genes, as detected by qRT-PCR and RNA-Seq (correlation coefficient = 0.853). This suggests that our RNA-Seq results are reliable.

## Discussion

Currently, with the development of improved detection techniques, a growing number of sperm-specific mRNAs have been identified. RNA-Seq technology has become an efficient and inexpensive method for the discovery of novel transcripts and genes. In our study, three ASM testes and three BSM testes were used as research samples, and 24,878 genes were screened by RNA-Seq. The results led to the identification of 16,237 genes that were upregulated and 8,641 genes that were downregulated during development. Using the next-generation platform, we found that most of the highly expressed genes are differentiation-related with identifiable roles in the development of the testis and spermatogenesis. Our analysis also identified related genes that were upregulated in ASM, such as *SPATA3*, *SPATA18, SPATA19, SPATA45, SPATC1, SPEM1, SPERT, SHBG, TEX35, TEX43* and *GSG1*. Among these genes, *SPATA3*, *SPATA18, SPATA19, SPATA45* and *SPATC1,* have known roles in spermatogenesis; *TEX35* and *TEX43* are associated with testis expression; *GSG1* is related to germ cell function; *SPEM1* influences spermatid maturation; and *SHBG* might regulate sex hormone-binding globulin. In addition, some novel genes detected are likely to be related to spermatogenesis.

Alternative splicing is a crucial mechanism for regulating gene expression and generating proteomic diversity. Recent estimates indicate that the expression of nearly 95% of human multi-exon genes involves AS [11, 12]. In metazoans, AS plays an important part in generating different protein products that function in diverse cellular processes, including cell growth, differentiation, and death. In our study, five kinds of AS events were detected, most of them involving SE. The SE events and the results of GO and KEGG analyses were comprehensively analyzed to determine the influence of AS events on the function of related genes [13]. In our study, IQ motif-containing G (*IQCG*) is regulated by 2 SE events as categorized under the GO terms “spermatogenesis,” “male gamete generation,” and “spermatid development”. The causality of the mutation was confirmed with a targeted null allele. Loss of *IQCG* disrupts spermiogenesis wherein tail formation is either incomplete or breaks apart from the sperm heads. Because IQ motif-containing genes typically regulate calmodulin, which in turn can impact the actin cytoskeleton, these findings suggest a potential role for localized calcium signaling in sperm flagellum morphogenesis, Ca^2+^ signaling is important for regulating sperm motility, most notably in the context of flagellar function and regulation [14–19]. 11 SE and 4 MXE events of the meiotic double-stranded break formation protein 1 (*MEI1*) gene also occurred in ASM. *MEI1* was upregulated in ASM and is related to the GO terms “spermatogenesis,” “male gamete generation,” and “spermatid development.” *MEI1* is expressed specifically in the testis and the first meiosis-specific mutation identified by forward genetic approaches in mammals. Mutations in the *MEI1* gene disrupt meiosis during spermatogenesis, especially in Americans of European descent [20–22].

Other enriched genes in this study that belong to the “spermatogenesis,” “male gamete generation,” and “spermatid development” GO terms include *SLC26A8, MEI1, EHMT2, DPY19L2, HIST1H2BA, CAPZA3, TSSK2, TSSK1B, KDM3A, ALMS1,* and *IQCG.* Testis anion transporter 1(Tat1) is a protein that, in humans, is encoded by the *SLC26A8* gene. *SLC26A8* gene was localized to developing spermatocytes [23–25]. The SLC26A8 protein specifically expressed in male germ cells and mature sperm. In the mouse, deletion of Tat1 caused male sterility as a results of lack of sperm motility, impaired sperm capacitation and structural defects of the flagella [26, 27]. It acts as a DIDS-sensitive anion exchanger that mediates chloride, sulfate, and oxalate transport. It also has critical anion exchange functions in male germ lines during meiosis, and therefore, may play a role in spermatogenesis [25]. The DPY19L2 protein, which belongs to the dpy-19 family, is highly expressed in testis and is required for sperm head elongation and acrosome formation during spermatogenesis. Mutations in *DPY19L2* are associated with an infertility disorder known as spermatogenic failure type 9 (SPGF9) [28–35]. Testis-specific serine kinase 2 (TSSK2), and Testis-specific serine kinase 1B (TSSK1B) are required during spermatid development. During spermatogenesis, *TSSK2* is highly expressed in the testis and is required for the transformation of a ring-shaped structure around the base of the flagellum that originates from the chromatoid body [36–41]. The above genes have well-established functions in spermatogenesis; however, the regulatory mechanisms in the horse have not been verified and thus require further investigation.

In our study, six upregulated KEGG pathways were detected. Though the “oocyte meiosis” pathway is named according to its role in female fertility, genes detected in the pathway also regulated meiosis of sperm. Meiosis plays a critical role in the formation of sperm. A total genes in the oocyte meiosis pathway were detected, with functions that influence meiosis I and II. Cell division cycle 25C (CDC25C), which encode threonine/tyrosine phosphatases related with in meiotic cell cycle regulation, were found to be differentially localized in rat testicular germ cells during spermatogenesis [42]. Aurora kinase a, also known as serine/threonine-protein kinase 6, is an enzyme that, in humans, is encoded by the *AURKA* gene and is a member of a family of mitotic serine/threonine kinases [43]. This gene is implicated to have an essential role during mitosis and meiosis [44], and its activity peaks during the G2 phase to M phase transition in the cell cycle [45]. The G2/mitotic-specific cyclin-B1 (CCNB1) is encoded by the *CCNB1* gene in humans [46]. The CCNE protein belongs to the highly conserved cyclin family, whose activity is required for cell cycle G1/S transition. This protein accumulates during the G1-S phase and is degraded as cells progress through the S-phase [47]. PLK1 is an early trigger for G2/M transition and supports the functional maturation of the centrosome in late G2/early prophase. PLK1 phosphorylates and activates components of the anaphase-promoting complex (APC). The function of APC can maintain cohesion of sister chromatids and anaphase inhibitors [48]. Meiotic recombination protein REC8 homolog is a meiosis-specific component of the cohesin complex that binds sister chromatids in preparation for the two divisions of meiosis [49, 50]. F-box only protein 43 (*FBXO43*), also called endogenous meiotic inhibitor 2 (*EMI2*), was originally identified in a yeast two-hybrid screen as a novel Xenopus polo-like kinase (Plx-1 ) [51, 52]. *FBXO43* is also essential for spermatocytes to complete meiotic divisions in mice. In male *FBXO43* knockout mice, spermatocytes cannot complete meiotic divisions, and spermatids are absent in the testes [53].

Our study focused on increasing understanding of the process of testis development and spermatogenesis in the Mongolian horse, which ultimately may lead to the development of approaches to improve fertility. Due to the lack of information available in regards to the genetics of horse spermatogenesis, we have inferred the function of certain DEGs according to available information in humans and mice. Furthermore, DEGs that have functions in development may not be directly applicable to many situations of infertility in the horse, such as azoospermia and asthenozoospermia.

## Conclusions

In our study, six mRNA libraries were constructed and sequenced from horse testes from ASM and BSM. A total of 16,237 upregulated and 8,641 downregulated genes were identified; most genes had functions within biological process categories and are known to participate in spermatogenesis and testis development. We analyzed a large number of genes related to spermatogenesis and development of the horse testis, including those that play important roles in different developmental stages. We also found that the AS events might influence the function of these genes and indirectly affect the spermatogenesis and development of the horse testis. These findings will help us to further understand how gene expression is regulated during testis development and spermatogenesis. Future research to evaluate the influence of AS events on testis development and spermatogenesis-related genes, or studies that combine other experimental results, such as miRNA and piRNA expression patterns, will provide more fundamental information in understanding the regulatory mechanisms of equine testis development and spermatogenesis at the molecular level.

## Materials and methods

### Ethics statement

This study was approved by and conducted according to the guidelines of the Institutional Animal Ethics Committee and Animal Care Guidelines of the Inner Mongolia Agricultural University. The animals did not unnecessarily suffer at any stage of this study.

### Animals and sample collection

We received permission to geld six healthy male Mongolian horses in Xilingol League, Inner Mongolia, China. The ages of the horses were determined based on physical examination of their teeth, as well as information from the owner. Three were sexually immature colts (before sexual maturation; samples BSM1–3), between 11 and 13 months old. The other three were sexually mature stallions, between 3 and 4 years old (after sexual maturation; samples ASM1–3). We surgically collected the testes of all six horses. The excised testes were stored in cryogenic vials with RNA/DNA sample protector (TaKaRa). Small aliquots (~ 5 g) from the testes of each animal from the parenchyma, including the seminiferous tubules and Leydig cells from the middle area, were immediately frozen in liquid nitrogen for quantitative real-time polymerase chain reaction (qRT-PCR) analysis. All horses were reared by their owner after our study.

### RNA quantification and qualification

Total RNA was extracted using TRIzol™ Reagent (Invitrogen) according to the manufacturer’s protocols and resuspended in nuclease-free water. Then, the samples were treated with DNase I (Invitrogen) to eliminate any contaminating genomic DNA. RNA degradation and contamination were monitored on 1% agarose gels. RNA purity was assessed using the NanoPhotometer® spectrophotometer (IMPLEN). RNA concentrations were measured using the Qubit® RNA Assay Kit with the Qubit® 2.0 Fluorometer (Life Technologies). RNA integrity was assessed using the RNA Nano 6000 Assay Kit with the Bioanalyzer 2100 system (Agilent Technologies).

### Library preparation for strand-specific transcriptome sequencing

Six libraries were constructed. The three immature testes libraries were designated BSM1, BSM2, and BSM3; and the three mature testes libraries were designated ASM1, ASM2, and ASM3. Approximately 3 μg RNA per sample were used as input material for the RNA sample preparations. Sequencing libraries were generated using NEBNext® Ultra™ Directional RNA Library Prep Kit for Illumina® (NEB) according to the manufacturer’s recommendations, and index codes were added to attribute sequences to each sample. Briefly, mRNA was purified from total RNA using poly-T oligo-conjugated magnetic beads. Fragmentation was conducted using divalent cations under elevated temperature in NEBNext First Strand Synthesis Reaction Buffer (5X). First-strand cDNA was synthesized using random hexamer primer and M-MuLV reverse transcriptase (RNaseH-). Second-strand cDNA synthesis was subsequently performed using DNA polymerase I and RNase H. In the reaction buffer, dNTPs with dTTP were replaced by dUTP. Remaining overhangs were converted into blunt ends via exonuclease/polymerase activity. After adenylation of the 3′ ends of the DNA fragments, NEBNext Adaptors with hairpin loop structure were ligated for hybridization. To select cDNA fragments of preferentially 150~200 bp in length, the libraries were purified with the AMPure XP system (Beckman Coulter). Then 3 μL of USER Enzyme (NEB) were used with size-selected, adaptor-ligated cDNA at 37 °C for 15 min, followed by 5 min at 95 °C before PCR. PCR was performed with Phusion High-Fidelity DNA polymerase, Universal PCR primers, and Index (X) Primer. Finally, the products were purified (AMPure XP system), and the library quality was assessed using the Agilent Bioanalyzer 2100 system.

### Clustering and sequencing

Clustering of the index-coded samples was performed on a cBot Cluster Generation System using the TruSeq PE Cluster Kit v3-cBot-HS (Illumina) according to the manufacturer’s instructions. After cluster generation, the library preparations were sequenced on an Illumina Hiseq platform and 150 bp paired-end reads were generated.

### Quality control

In order to guarantee data quality in our analysis, a Perl script was used to filter the Raw Data. The steps were as follows: The clean data were obtained by removing reads containing adapter, reads containing ploy-N and low quality reads from raw data.

### Reads mapping to the reference genome

The reference genome and gene model annotation files were directly downloaded from the www.ensembl.org. The index of the reference genome was built using Bowtie v2.2.3, and paired-end clean reads were aligned to the reference genome using TopHat v2.0.12 [54, 55]. TopHat was selected as the mapping tool because it can generate a database of splice junctions based on the gene model annotation file and thus provides a better mapping result than other non-splice mapping tools [56–58].

### Quantification of gene expression

HTSeq v0.6.1 was used to count the read numbers mapped to each gene [59]. Then, the expected number of Fragments Per Kilobase of transcript sequence per Million base pairs sequenced (FPKM) of each gene was calculated based on the length of the gene and read count mapped to the gene. The FPKM simultaneously considers the effect of the sequencing depth and gene length for the read count, and is currently the most commonly used method for estimating gene expression levels [60].

### Differential expression analysis

Differential expression analysis of two stages (three biological replicates per stage) was performed using the DESeq R package (1.18.0). DESeq provides statistical routines for determining differential expression within digital gene expression data using a model based on the negative binomial distribution. The resulting *P*-values were adjusted using the Benjamin and Hochberg’s approach for controlling the false discovery rate. Genes with an adjusted *P*-value < 0.05 found by DESeq were assigned as differentially expressed [61, 62].

### GO and KEGG enrichment analyses of DEGs

GO enrichment analysis of DEGs was implemented by the GOseq R package, in which the gene length bias was corrected. GO terms with corrected *P*-value < 0.05 were considered significantly enriched DEGs [63]. KEGG is a database resource for understanding high-level functions and utilities of biological systems, such as the cell, the organism and the ecosystem, based on molecular-level information, especially large-scale molecular datasets generated by genome sequencing and other high-throughput experimental technologies (http://www.genome.jp/kegg/) [64]. We used KOBAS software to test the statistical enrichment of DEGs in the KEGG pathways [65].

### Differential gene transcription factor analysis

Putative transcription factors were analyzed using the animal transcription factor database AnimalTFDB 2.0 [66]. For genes included in the database, the transcription factors were directly screened by Ensembl geneID; Non-Ensembl geneID genes were screened through the horse transcription factor protein sequence database by BLASTX.

### Novel transcript prediction and alternative splicing analysis

The Cufflinks v2.1.1 Reference Annotation Based Transcript (RABT) assembly method was used to construct and identify both known and novel transcripts from TopHat alignment results. Alternative splicing events were classified by the software rMATS (http://rnaseq-mats.sourceforge.net/index.html). The number of AS events in each sample was estimated separately.

### qRT-PCR validation

A piece of testis was excised and frozen immediately in liquid nitrogen for qRT-PCR analysis. Total RNA was extracted using TRIzol™ Reagent (Invitrogen) according to the manufacturer’s protocols. About 0.5 μg of total RNA was used as template to synthesize first-strand cDNA using a miScript II RT Kit (QIAGEN) according to the manufacturer’s protocols. The resultant cDNA was diluted to 0.1 μg/μL for further analysis by the MX3000P Real-Time PCR System (Agilent Technologies) using SYBR® Premix Ex Taq™ II(TaKaRa). β-actin was chosen as an internal reference gene to eliminate sample-to-sample variations based on evidence of its role as a constitutive house-keeping gene for qRT-PCR and its use in normalizing changes in specific gene expressions [67]. The relative gene expression levels were calculated using the 2^−ΔΔCt^ method. The DEGs between ASM and BSM testis were analyzed by t-test.

## Supplementary information

**Additional file 1: Table S1.** List of data output quality.

**Additional file 2: Table S2.** Alignment of Reads and reference genome.

**Additional file 3: Table S3.** Classification and quantity statistics of AS events.

**Additional file 4: Table S4.** The results of qRT-PCR validation.

## Data Availability

The accession number for the raw and processed profiling by high-throughput RNA sequencing technology reported in this paper is GEO: GSE101697.

## References

[1] Hecht NB (1998). Molecular mechanisms of male germ cell differentiation. BioEssays.

[2] Eddy EM, O'Brien DA (1998). Gene expression during mammalian meiosis. Curr Top Dev Biol.

[3] Mortazavi A, Williams BA, McCue K, Schaeffer L, Wold B (2008). Mapping and quantifying mammalian transcriptomes by RNA-Seq. Nat Methods.

[4] Marioni JC, Mason CE, Mane SM, Stephens M, Gilad Y (2008). RNA-seq: an assessment of technical reproducibility and comparison with gene expression arrays. Genome Res.

[5] Wang Z, Gerstein M, Snyder M (2010). RNA-Seq: a revolutionary tool for transcriptomics. Nat Rev Genet.

[6] Ramsköld D, Wang ET, Burge CB, Sandberg R (2009). An abundance of ubiquitously expressed genes revealed by tissue transcriptome sequence data. PLoS Comput Biol.

[7] Djureinovic D, Fagerberg L, Hallstrom B, Danielsson A, Lindskog C, Uhlen M, Ponten F (2014). The human testis-specific proteome defined by transcriptomics and antibody-based profiling. Mol Hum Reprod.

[8] Anand M, Prasad BV (2012). The computational analysis of human testis transcriptome reveals closer ties to pluripotency. J Hum Reprod Sci.

[9] Jun J, Cho YS, Hu H, Kim H-M, Jho S, Gadhvi P, Park KM, Lim J, Paek WK, Han K (2014). Whole genome sequence and analysis of the Marwari horse breed and its genetic origin. BMC Genomics.

[10] Doan R, Cohen ND, Sawyer J, Ghaffari N, Johnson CD, Dindot SV (2012). Whole-genome sequencing and genetic variant analysis of a quarter horse mare. BMC Genomics.

[11] Pan Q, Shai O, Lee LJ, Frey BJ, Blencowe BJ. Deep surveying of alternative splicing complexity in the human transcriptome by high-throughput sequencing. Nat Genet. 2008;40(12):1413–5. 10.1038/ng.259 18978789

[12] Wahl MC, Will CL, Lührmann R (2009). The Spliceosome: design principles of a dynamic RNP machine. Cell.

[13] Chen M, Manley JL (2009). Mechanisms of alternative splicing regulation: insights from molecular and genomics approaches. Nat Rev Mol Cell Biol.

[14] Ignotz GG, Suarez SS (2005). Calcium/calmodulin and calmodulin kinase II stimulate hyperactivation in demembranated bovine sperm. Biol Reprod.

[15] Harris TP, Schimenti KJ, Munroe RJ, Schimenti JC (2014). IQ motif-containing G (Iqcg) is required for mouse spermiogenesis. G3 (Bethesda).

[16] Marquez B, Ignotz G, Suarez SS (2007). Contributions of extracellular and intracellular Ca2+ to regulation of sperm motility: release of intracellular stores can hyperactivate CatSper1 and CatSper2 null sperm. Dev Biol.

[17] Lindemann CB, Goltz JS, Kanous KS. Regulation of activation state and flagellar wave form in epididymal rat sperm: evidence for the involvement of both Ca2+ and cAMP. Cell Motil Cytoskeleton. 1987;8(4):324–32. 10.1002/cm.970080405 2826020

[18] Ho H-C, Granish KA, Suarez SS. Hyperactivated motility of bull sperm is triggered at the axoneme by Ca2+ and not cAMP. Dev Biol. 2002;250(1):208–17. 10.1006/dbio.2002.0797 12297107

[19] Darszon A, Nishigaki T, Beltran C, Treviño CL. Calcium channels in the development, maturation, and function of spermatozoa. Physiol Rev. 2011;91(4):1305–55. 10.1152/physrev.00028.2010 22013213

[20] Sato H, Miyamoto T, Yogev L, Namiki M, Koh E, Hayashi H, Sasaki Y, Ishikawa M, Lamb DJ, Matsumoto N (2006). Polymorphic alleles of the human MEI1 gene are associated with human azoospermia by meiotic arrest. J Hum Genet.

[21] Reinholdt LG, Schimenti JC (2005). Mei1 is epistatic to Dmc1 during mouse meiosis. Chromosoma.

[22] Libby BJ, Reinholdt LG, Schimenti JC (2003). Positional cloning and characterization of Mei1, a vertebrate-specific gene required for normal meiotic chromosome synapsis in mice. Proc Natl Acad Sci.

[23] Lohi H, Kujala M, Mäkelä S, Lehtonen E, Kestilä M, Saarialho-Kere U, Markovich D, Kere J (2002). Functional characterization of three novel tissue-specific anion exchangers SLC26A7, −A8, and -A9. J Biol Chem.

[24] Dirami T, Rode B, Jollivet M, Da Silva N, Escalier D, Gaitch N, Norez C, Tuffery P, Wolf J-P, Becq F (2013). Missense mutations in SLC26A8, encoding a sperm-specific activator of CFTR, are associated with human asthenozoospermia. Am J Hum Genet.

[25] Toure A, Morin L, Pineau C, Becq F, Dorseuil O, Gacon G. Tat1, a novel sulfate transporter specifically expressed in human male germ cells and potentially linked to rhogtpase signaling. J Biol Chem. 2001;276(23):20309–15. 10.1074/jbc.M011740200 11278976

[26] Hernández-González EO, Treviño CL, Castellano LE, de la Vega-Beltrán JL, Ocampo AY, Wertheimer E, Visconti PE, Darszon A. Involvement of cystic fibrosis transmembrane conductance regulator in mouse sperm capacitation. J Biol Chem. 2007;282(33):24397–406. 10.1074/jbc.M701603200 17588945

[27] Rode B, Dirami T, Bakouh N, Rizk-Rabin M, Norez C, Lhuillier P, Lores P, Jollivet M, Melin P, Zvetkova I (2012). The testis anion transporter TAT1 (SLC26A8) physically and functionally interacts with the cystic fibrosis transmembrane conductance regulator channel: a potential role during sperm capacitation. Hum Mol Genet.

[28] Ghédir H, Ibala-Romdhane S, Okutman O, Viot G, Saad A, Viville S (2016). Identification of a new DPY19L2 mutation and a better definition of DPY19L2 deletion breakpoints leading to globozoospermia. Mol Hum Reprod.

[29] Coutton C, Abada F, Karaouzene T, Sanlaville D, Satre V, Lunardi J, Jouk P-S, Arnoult C, Thierry-Mieg N, Ray PF (2013). Fine characterisation of a recombination hotspot at the DPY19L2 locus and resolution of the paradoxical excess of duplications over deletions in the general population. PLoS Genet.

[30] Zhu F, Gong F, Lin G, Lu G (2013). DPY19L2 gene mutations are a major cause of globozoospermia: identification of three novel point mutations. Mol Hum Reprod.

[31] ElInati E, Kuentz P, Redin C, Jaber S, Vanden Meerschaut F, Makarian J, Koscinski I, Nasr-Esfahani MH, Demirol A, Gurgan T (2012). Globozoospermia is mainly due to DPY19L2 deletion via non-allelic homologous recombination involving two recombination hotspots. Hum Mol Genet.

[32] Coutton C, Zouari R, Abada F, Ben Khelifa M, Merdassi G, Triki C, Escalier D, Hesters L, Mitchell V, Levy R (2012). MLPA and sequence analysis of DPY19L2 reveals point mutations causing globozoospermia. Hum Reprod.

[33] Koscinski I, ElInati E, Fossard C, Redin C, Muller J, Velez de la Calle J, Schmitt F, Ben Khelifa M, Ray P, Kilani Z (2011). DPY19L2 deletion as a major cause of globozoospermia. Am J Hum Genet.

[34] Harbuz R, Zouari R, Pierre V, Ben Khelifa M, Kharouf M, Coutton C, Merdassi G, Abada F, Escoffier J, Nikas Y, Vialard F, Koscinski I, Triki C, Sermondade N, Schweitzer T, Zhioua A, Zhioua F, Latrous H, Halouani L, Ouafi M, Makni M, Jouk PS, Sèle B, Hennebicq S, Satre V, Viville S, Arnoult C, Lunardi J, Ray PF. A recurrent deletion of DPY19L2 causes infertility in man by blocking sperm head elongation and acrosome formation. Am J Hum Genet. 2011;88(3):351–61. 10.1016/j.ajhg.2011.02.007 PMC3059422 21397064

[35] Yassine S, Escoffier J, Martinez G, Coutton C, Karaouzène T, Zouari R, Ravanat J-L, Metzler-Guillemain C, Lee HC, Fissore R (2015). Dpy19l2-deficient globozoospermic sperm display altered genome packaging and DNA damage that compromises the initiation of embryo development. Mol Hum Reprod.

[36] Zhang Z, Shen X, Jones BH, Xu B, Herr JC, Strauss JF 3rd. Phosphorylation of mouse sperm axoneme central apparatus protein SPAG16L by a testis-specific kinase, TSSK2. Biol Reprod. 2008;79(1):75–83. 10.1095/biolreprod.107.066308 PMC2660405 18367677

[37] Xu B, Hao Z, Jha KN, Zhang Z, Urekar C, Digilio L, Pulido S, Strauss JF, Flickinger CJ, Herr JC (2008). Targeted deletion of Tssk1 and 2 causes male infertility due to haploinsufficiency. Dev Biol.

[38] Li Y, Sosnik J, Brassard L, Reese M, Spiridonov NA, Bates TC, Johnson GR, Anguita J, Visconti PE, Salicioni AM (2011). Expression and localization of five members of the testis-specific serine kinase (Tssk) family in mouse and human sperm and testis. Mol Hum Reprod.

[39] Shang P, Hoogerbrugge J, Baarends W, Grootegoed JA (2013). Evolution of testis-specific kinases TSSK1B and TSSK2 in primates. Andrology.

[40] Shang P, Baarends WM, Hoogerbrugge J, Ooms MP, van Cappellen WA, de Jong AA, Dohle GR, van Eenennaam H, Gossen JA, Grootegoed JA. Functional transformation of the chromatoid body in mouse spermatids requires testis-specific serine/threonine kinases. J Cell Sci. 2010;123(Pt 3):331–9. 10.1242/jcs.059949 20053632

[41] Kueng P, Nikolova Z, Djonov V, Hemphill A, Rohrbach V, Boehlen D, Zuercher G, Andres AC, Ziemiecki A. A novel family of serine/threonine kinases participating in spermiogenesis. J Cell Biol. 1997;139(7):1851–9. 10.1083/jcb.139.7.1851 PMC2132653 9412477

[42] Mizoguchi S, Kim KH. Expression of cdc25 phosphatases in the germ cells of the rat testis. Biol Reprod. 1997;56(6):1474–81. 10.1095/biolreprod56.6.1474 9166700

[43] Zhou H, Kuang J, Zhong L, Kuo WL, Gray JW, Sahin A, Brinkley BR, Sen S. Tumour amplified kinase STK15/BTAK induces centrosome amplification, aneuploidy and transformation. Nat Genet. 1998;20(2):189–93. 10.1038/2496 9771714

[44] Crane R, Gadea B, Littlepage L, Wu H, Ruderman JV. Aurora A, meiosis and mitosis. Biol Cell. 2004;96(3):215–29. 10.1016/j.biolcel.2003.09.008 15182704

[45] Bischoff JR, Plowman GD. The Aurora/Ipl1p kinase family: regulators of chromosome segregation and cytokinesis. Trends Cell Biol. 1999;9(11):454–9. 10.1016/s0962-8924(99)01658-x 10511710

[46] Sartor H, Ehlert F, Grzeschik KH, Müller R, Adolph S. Assignment of two human cell cycle genes, CDC25C and CCNB1, to 5q31 and 5q12, respectively. Genomics. 1992;13(3):911–2. 10.1016/0888-7543(92)90190-4 1386342

[47] Ohtsubo M, Theodoras AM, Schumacher J, Roberts JM, Pagano M. Human cyclin E, a nuclear protein essential for the G1-to-S phase transition. Mol Cell Biol. 1995;15(5):2612–24. 10.1128/mcb.15.5.2612 PMC230491 7739542

[48] van de Weerdt BCM, Medema RH (2006). Polo-like kinases: a team in control of the division. Cell Cycle.

[49] Xu H, Beasley MD, Warren WD, van der Horst GT, McKay MJ. Absence of mouse REC8 cohesin promotes synapsis of sister chromatids in meiosis. Dev Cell. 2005;8(6):949–61. 10.1016/j.devcel.2005.03.018 15935783

[50] Lee J, Iwai T, Yokota T, Yamashita M (2003). Temporally and spatially selective loss of Rec8 protein from meiotic chromosomes during mammalian meiosis. J Cell Sci.

[51] Schmidt A, Rauh NR, Nigg EA, Mayer TU. Cytostatic factor: an activity that puts the cell cycle on hold. J Cell Sci. 2006;119(Pt 7):1213-8. 10.1242/jcs.02919 16554437

[52] Tung JJ, Hansen DV, Ban KH, Loktev AV, Summers MK, Adler JR 3rd, Jackson PK. A role for the anaphase-promoting complex inhibitor Emi2/XErp1, a homolog of early mitotic inhibitor 1, in cytostatic factor arrest of Xenopus eggs. Proc Natl Acad Sci U S A. 2005;102(12):4318–23. 10.1073/pnas.0501108102 PMC552977 15753281

[53] Gopinathan L, Szmyd R, Low D, Diril MK, Chang HY, Coppola V, Liu K, Tessarollo L, Guccione E, van Pelt AMM7, Kaldis P. Emi2 is essential for mouse spermatogenesis. Cell Rep. 2017;20(3):697–708. 10.1016/j.celrep.2017.06.033 PMC7974383 28723571

[54] Langmead B, Trapnell C, Pop M, Salzberg SL (2009). Ultrafast and memory-efficient alignment of short DNA sequences to the human genome. Genome Biol.

[55] Langmead B, Salzberg SL (2012). Fast gapped-read alignment with Bowtie 2. Nat Methods.

[56] Trapnell C, Pachter L, Salzberg SL (2009). TopHat: discovering splice junctions with RNA-Seq. Bioinformatics.

[57] Kim D, Pertea G, Trapnell C, Pimentel H, Kelley R, Salzberg SL (2013). TopHat2: accurate alignment of transcriptomes in the presence of insertions, deletions and gene fusions. Genome Biol.

[58] Trapnell C, Roberts A, Goff L, Pertea G, Kim D, Kelley DR, Pimentel H, Salzberg SL, Rinn JL, Pachter L (2012). Differential gene and transcript expression analysis of RNA-seq experiments with TopHat and Cufflinks. Nat Protoc.

[59] Anders S. HTSeq: analysing high-throughput sequencing data with Python. 2010. 10.1093/bioinformatics/btac166 PMC9113351 35561197

[60] Trapnell C, Williams BA, Pertea G, Mortazavi A, Kwan G, van Baren MJ, Salzberg SL, Wold BJ, Pachter L (2010). Transcript assembly and quantification by RNA-Seq reveals unannotated transcripts and isoform switching during cell differentiation. Nat Biotechnol.

[61] Anders S, Huber W (2010). Differential expression analysis for sequence count data. Genome Biol.

[62] Anders S, Huber W. Differential expression of RNA-Seq data at the gene level-the DESeq package. 2012.

[63] Young MD, Wakefield MJ, Smyth GK, Oshlack A (2010). Gene ontology analysis for RNA-seq: accounting for selection bias. Genome Biol.

[64] Kanehisa M, Araki M, Goto S, Hattori M, Hirakawa M, Itoh M, Katayama T, Kawashima S, Okuda S, Tokimatsu T (2008). KEGG for linking genomes to life and the environment. Nucleic Acids Res.

[65] Mao X, Cai T, Olyarchuk JG, Wei L (2005). Automated genome annotation and pathway identification using the KEGG Orthology (KO) as a controlled vocabulary. Bioinformatics.

[66] Zhang H-M, Liu T, Liu C-J, Song S, Zhang X, Liu W, Jia H, Xue Y, Guo A-Y (2015). AnimalTFDB 2.0: a resource for expression, prediction and functional study of animal transcription factors. Nucleic Acids Res.

[67] Mori R, Wang Q, Danenberg K, Pinski J, Danenberg P (2008). Both β-actin and GAPDH are useful reference genes for normalization of quantitative RT-PCR in human FFPE tissue samples of prostate cancer. Prostate.

