# Supplementary material for: Transcriptome profiling of developing testes and spermatogenesis in the Mongolian horse
Source: BMC Genet. 2020 Apr 28;21:46. doi: 10.1186/s12863-020-00843-5 (PMC7187496; doi:10.1186/s12863-020-00843-5)
Supplement: Supplementary file 1 — Additional file 1: Table S1. List of data output quality. [file 12863_2020_843_MOESM1_ESM.docx]

Table S1 List of data output quality

| Sample name | Raw reads | Clean reads | Error rate(%) | Q20(%) | Q30(%) | GC content(%) |
| --- | --- | --- | --- | --- | --- | --- |
| BS1 | 58277598 | 57482858 | 0.01 | 98.37 | 95.91 | 48.89 |
| BS2 | 59921568 | 58972988 | 0.01 | 98.45 | 96.1 | 48.46 |
| BS3 | 53371478 | 52475320 | 0.01 | 98.48 | 96.17 | 48.98 |
| AS1 | 59523578 | 58411056 | 0.01 | 98.43 | 96.03 | 49.97 |
| AS2 | 61727216 | 60775868 | 0.01 | 98.33 | 95.81 | 49.6 |
| AS3 | 53956064 | 53135736 | 0.01 | 98.38 | 95.91 | 49.6 |

Note:

1. Raw reads：Statistical raw sequence data

2. Clean reads: Statistics for the filtered data

3. Q20、Q30: Calculate the percentage of the total base of Phred value greater than 20 and 30, respectively

4. GC content: Calculate the total number of base G and C and the percentage of total base number
