# Supplementary material for: Transcriptome profiling of developing testes and spermatogenesis in the Mongolian horse
Source: BMC Genet. 2020 Apr 28;21:46. doi: 10.1186/s12863-020-00843-5 (PMC7187496; doi:10.1186/s12863-020-00843-5)
Supplement: Supplementary file 2 — Additional file 2: Table S2. Alignment of Reads and reference genome. [file 12863_2020_843_MOESM2_ESM.docx]

Table S2 Alignment of Reads and reference genome

| Sample name | BS1 | BS2 | BS3 | AS1 | AS2 | AS3 |
| --- | --- | --- | --- | --- | --- | --- |
| Total reads | 57482858 | 58972988 | 52475320 | 58411056 | 60775868 | 53135736 |
| Total mapped | 49544664 (86.19%) | 50977489 (86.44%) | 44970066 (85.7%) | 50154994 (85.87%) | 52106893 (85.74%) | 45639892 (85.89%) |
| Multiple mapped | 769537 (1.34%) | 820207 (1.39%) | 757219 (1.44%) | 865718 (1.48%) | 967532 (1.59%) | 881231  (1.66%) |
| Uniquely mapped | 48775127 (84.85%) | 50157282 (85.05%) | 44212847 (84.25%) | 49289276 (84.38%) | 51139361 (84.14%) | 44758661 (84.23%) |
| Read-1 | 24956766 (43.42%) | 25616683 (43.44%) | 22540529 (42.95%) | 25259215 (43.24%) | 26257895 (43.2%) | 22962720 (43.22%) |
| Read-2 | 23818361 (41.44%) | 24540599 (41.61%) | 21672318 (41.3%) | 24030061 (41.14%) | 24881466 (40.94%) | 21795941 (41.02%) |
| Reads map to '+' | 24358384 (42.38%) | 25055369 (42.49%) | 22098091 (42.11%) | 24639652 (42.18%) | 25572721 (42.08%) | 22376022 (42.11%) |
| Reads map to '-' | 24416743 (42.48%) | 25101913 (42.57%) | 22114756 (42.14%) | 24649624 (42.2%) | 25566640 (42.07%) | 22382639 (42.12%) |
| Non-splice reads | 32691913 (56.87%) | 34028728 (57.7%) | 30088902 (57.34%) | 28163504 (48.22%) | 29933197 (49.25%) | 26300556 (49.5%) |
| Splice reads | 16083214 (27.98%) | 16128554 (27.35%) | 14123945 (26.92%) | 21125772 (36.17%) | 21206164 (34.89%) | 18458105 (34.74%) |

Note:

1. Total reads：Statistics for the filtered data

2. Total mapped：The number of sequences that can be localized to the genome;

3 Multiple mapped：The number of sequencing sequences with multiple alignment positions on a reference sequence;

4. Uniquely mapped：The number of sequences in a reference sequence with a unique alignment.

5. Reads map to '+'，Reads map to '-'：The alignment of sequence to positive and negative chains in genome.

6. Splice reads: The reads mapped to the border of two exons, also called junction reads.

Non-splice reads: The reads for the entire sequence are mapped to one exon.
