# Supplementary material for: Transcriptome profiling of developing testes and spermatogenesis in the Mongolian horse
Source: BMC Genet. 2020 Apr 28;21:46. doi: 10.1186/s12863-020-00843-5 (PMC7187496; doi:10.1186/s12863-020-00843-5)
Supplement: Supplementary file 3 — Additional file 3: Table S3. Classification and quantity statistics of AS events. [file 12863_2020_843_MOESM3_ESM.docx]

Table S3 Classification and quantity statistics of AS events

| Event Type | NumEvents JC.only | SigEvents.JC.only | NumEvents JC.only+reads On Target | SigEvents.JC.only+  reads On Target |
| --- | --- | --- | --- | --- |
| SE | 16702 | 987(431:556) | 16724 | 991(434:557) |
| MXE | 1888 | 341(172:169) | 1891 | 311(163:148) |
| A5SS | 43 | 2(0:2) | 43 | 2(0:2) |
| A3SS | 75 | 6(4:2) | 76 | 6(4:2) |
| RI | 40 | 1(1:0) | 40 | 1(1:0) |

Note:

1.Event Type: AS event type (SE, MXE, A5SS, A3SS, RI)

2. NumEvents JC.only: Total number of differential AS events. Only use junction counts to AS event detection

3. SigEvents.JC.only: Total number of differential AS events.

4. NumEvents JC.only+reads On Target: Total number of differential AS events. Simultaneous use junction counts and reads on targent to AS event detection

5. SigEvents.JC.only+reads On Target: Total number of differential AS events, meaning as (3)
